# Autologous Matrix of Platelet-Rich Fibrin in Wound Care Settings: A Systematic Review of Randomized Clinical Trials

**DOI:** 10.3390/jfb11020031

**Published:** 2020-05-14

**Authors:** Chayane Karla Lucena de Carvalho, Beatriz Luci Fernandes, Mauren Abreu de Souza

**Affiliations:** Graduate Program on Health Technology, Pontifical Catholic University of Paraná, Curitiba, PR 80215-901, Brazil; chaycarvalho7@gmail.com (C.K.L.d.C.); mauren.souza@pucpr.br (M.A.d.S.)

**Keywords:** platelet-rich fibrin, wound healing, skin wounds, wound dressing

## Abstract

Platelet-rich fibrin (PRF) consists of a matrix that provides the necessary elements for wound healing, acting as a biodegradable scaffold for cell migration, proliferation, and differentiation, in addition to the delivery of growth factors and angiogenesis. This study aims to determine the effectiveness of the autologous PRF in the treatment of wounds of different etiologies. We carried out a systematic review of randomized clinical trials, guided by the recommendations of the Cochrane Collaboration using the following databases: Pubmed/MEDLINE, EMBASE, Web of Science, and CENTRAL. The search strategy resulted in the inclusion of ten studies that evaluated the use of PRF dressings for the healing of acute or chronic wounds of multiple etiologies. Among the 172 participants treated with PRF in wounds of varying etiologies and different segment times, 130 presented favorable events with the use of the intervention. Among the 10 studies included, only two of them did not demonstrate better results than the control group. The studies showed clinical heterogeneity, making it impossible to perform a meta-analysis. The findings do not provide enough evidence to support the routine use of PRF dressings as the first line of treatment for the healing of acute or chronic wounds of different etiologies. There was great variability in the application of the various protocols and the ways to prepare the PRF, resulting in clinical heterogeneity. Therefore, it makes it impossible to synthesize and to collect evidence from different types of studies in the meta-analysis, which affects the results and their proper discussion.

## 1. Introduction

Human skin is an organ structured by many tissues, designed to develop multiple functions such as thermoregulation, vitamin D metabolization, detection of sensory stimuli, as well as reacting to mechanical trauma, chemical reagents, and pathogens [1,2]. However, to maintain its functionality, it is necessary to preserve its structural integrity.

Ruptures of the skin layers or adjacent tissues, called wounds, bring anatomical and functional changes, resulting in increased morbidity with a high impact on the public health sectors [3]. Wounds have a huge financial burden on health systems around the world. For example, they account for more than US$ 25 billion per year in the USA, due to the expenses with therapies, which are sometimes ineffective [4]. 

The establishment of wounds signals to the body the immediate need to correct the lesions through the self-regenerative process known as healing [5]. Wound healing is a physiological process including a cascade of complex, orderly and interconnected events, involving many types of cells interacting in a highly sophisticated temporal sequence, guided by the release of soluble mediators and signals that can influence the direction of the circulating cells to the damaged tissues [6,7].

Over the years, traditional and well-established medicine has been improved, giving rise to regenerative types of medicines. This new approach involves the use of therapeutic alternatives based on the human body itself (i.e., autologous or heterologous). In the context of wound healing, the regenerative technologies are available based on a variety of mechanisms, such as vascular fraction, the stroma of adipose tissue, platelet-rich plasmas, and bone marrow concentration, among others. In this scenario, these options help to reduce the economic and psychosocial burden, usually generated by chronic and highly complex wounds, including ulcerations, surgical, and necrotic wounds as well [8,9].

In this scenario, autologous platelet biomaterials represent an important source of cytokines and growth factors widely used for clinical and surgical applications, including tissue regeneration and wound healing [10]. Among these approaches, platelet-rich fibrin (PRF) is considered the second-generation of platelet concentrate, developed in France in 2001 [11].

The use of emerging cellular therapeutic technologies, such as platelet biomaterials, covers many pathologies as a therapeutic agent and/or coadjuvant treatment, resulting in improvements in the quality of life of the patients [12].

The first platelet concentrates were introduced in 1998. The platelet-rich plasma (PRP) was primarily used in odontology and later in otorhinolaryngology and orthopedics, to accelerate tissue regeneration, especially for soft ones. However, there is a lack of uniformity in the methods of obtaining PRP and the use, or non-use, of bovine thrombin for its activation [13].

Platelet-rich fibrin (PRF), considered a second-generation platelet concentrate, was developed in France in 2001 [11]. It does not need biochemical additives, such as anticoagulants or bovine thrombin. It is possible to obtain the PRF from the controlled centrifugation of the venous blood itself, mainly because of the soluble fibrinogen found in the fibrin, which is responsible to polymerize it in a tridimensional structure [14].

PRF consists of a matrix in which cytokines, growth factors, and platelet cells are retained and can be constantly released, offering the necessary elements for wound healing, acting as a biodegradable scaffold for the delivery of growth factors, collagen synthesis, and angiogenesis. Its applicability is observed in various health fields [15,16,17]. Its autologous origin and immediate availability are also noteworthy, as well as the factors related to safety, costs, and practical aspects, such as short manufacturing and implementation time [18]. Some growth factors and cytokines, mainly generated from platelets and leukocytes in the fibrin clot during wound healing, regulate some important biological processes like cell migration and differentiation, angiogenesis, and extracellular matrix synthesis [19].

Fibrin membrane (FM) is a natural biopolymer with an important capacity for the regeneration of several injured tissues [20]. The adhesive FM, when arranged on the wound bed, changes its configuration and mechanical properties over time because of the fibrin matrix retraction and expression of the secretome mostly containing the vital signaling molecules. The FM can be combined with secondary dressings, including alginates, hydrocolloids, and gauze, enhancing the action of the biomaterial [21]. Therefore, the PRF-based dressings accelerate the healing of hard and soft tissues and can be used in the treatment of different types of lesions [20,22,23].

The preparation protocol for the FM consists of blood collection through the venous puncture and subsequent centrifugation in tubes without anticoagulant, in order to form a strong polymerized fibrin clot [24]. The fibrin clot, a natural polymer, is one of the three layers resulting from the centrifugation process presenting about 97% of platelets and 50% of leukocytes from the initial blood volume, which is incorporated and distributed in a tridimensional way [25].

Regenerative medicine has especially evolved, aimed at skin healing, with different approaches: (1) individual application of skin cells, (2) biopolymer scaffolding, and (3) with the combination of both—which is classified as acellular scaffolding (i.e., temporary skin substitutes with allogeneic or autologous epithelial cells). Their final purpose is for the treatment and healing of both acute and chronic skin wounds, contributing to a reduction in the morbidity and mortality of the affected population [21,26].

Although there have been technological advances in wound treatments, innovative approaches using natural biopolymers with higher effectiveness and lower costs need further clinical studies. Therefore, the main purpose of this study is to provide information from available literature to analyze the effectiveness of autologous FM in the treatment of wounds of varying etiologies. 

## 2. Materials and Methods 

This paper presents a systematic review of randomized clinical trials, guided by the Cochrane Collaboration recommendations contained in the Handbook for Systematic Reviews of Interventions, version 6.0 [27], and described by the Preferred Reporting Items for Systematic Reviews and Meta-Analyses (PRISMA) [28].

The systematic review is a type of secondary study conducted from a defined research question to identify, evaluate, select, and synthesize pieces of evidence from primary studies that meet the predefined eligibility criteria [27].

We used the acronym PICO (Patient, Intervention, Comparison and Outcomes) to elaborate the research question, in which we related “P” to wounds of any etiology; “I” to the autologous matrix of platelet-rich fibrin (in different formulations, concentrations, and modes of obtention); “C” to different dressings technologies; and “O” to healing, reduction of the wound area and adverse events. Therefore, the research question was: what is the effectiveness of dressing based on the autologous matrix of platelet-rich fibrin for the treatment of wounds of different etiologies, when compared to other dressings technologies for the healing outcomes and reduction of the wound area? 

In this study, we included randomized clinical trials with any size, where the autologous matrix of PRF was adopted, including at least one of the groups treated to achieve the proposed outcome. We excluded studies involving periodontal proceduresm, and interventions not limited to the use of PRF and the use of heterologous materials.

We recovered the relevant studies through a search strategy in databases: Medical Literature Analysis and Retrieval System Online/Pubmed (MEDLINE); Excerpta Medica Database (EMBASE); Web of Science; and the Cochrane Central Register of Controlled Trials (CENTRAL). The keywords and the searching strategies used in each database are presented in Table 1.

Additionally, a manual search was conducted by gray literature, which consists of studies not controlled by scientific editors, such as government reports, thesis, dissertations, and abstracts published in conference proceedings. We evaluated the reference list of clinical trials in order to identify not eligible studies, i.e., the ones not included in the searching strategies.

We selected descriptors and their synonyms for the search of primary studies in previously established databases: Medical Subject Headings (MeSH)—platelet-rich fibrin OR autologous platelet-rich fibrin OR platelet-rich fibrin matrix; AND wound AND randomized controlled trial.

All the recovered citations were screened and evaluated for their eligibility according to the inclusion criteria by two independent reviewers. The screening and selection process included two phases: the evaluation of the titles and abstracts of all identified studies, fully reading the selected studies, and making a justification for the exclusions.

We performed a critical analysis of the included studies using the Cochrane Risk of Bias Tool to assess the risk of bias, available in the Review Manager, version 5.3. The two reviewers judged the studies according to three categories: low risk of bias, high risks of bias and undetermined risk of bias for the generation of the domain of the randomization of the samples by allocation sequence (selected bias); blinding of participants and researchers (performance bias); blinding of outcomes evaluators (detection bias); systematic differences in segment losses (frictional bias); incomplete outcomes or selective report of outcomes (report bias). The Kappa coefficient determined the level of agreement between the reviewers on the inclusion or exclusion of the analyzed studies. A third reviewer re-evaluated the divergences.

We organized the data from these studies in a narrative synthesis presentation form, including authorship and year of publication, country of origin, the title of the manuscript and the journal, clinical information such as the number of participants, intervention groups and comparison between them, intervention time, and main outcomes.

We categorized all included studies through allocation confidentiality, according to the Cochrane Handbook, as described: category A—the allocation process was adequately described; category B—although the allocation process has not been described, the study points out to randomization; category C—allocation confidentiality was conducted improperly (for example, arrival order, medical record number, and date of birth); category D—the randomization of the participants was not demonstrated.

## 3. Results

**Figure 1:**
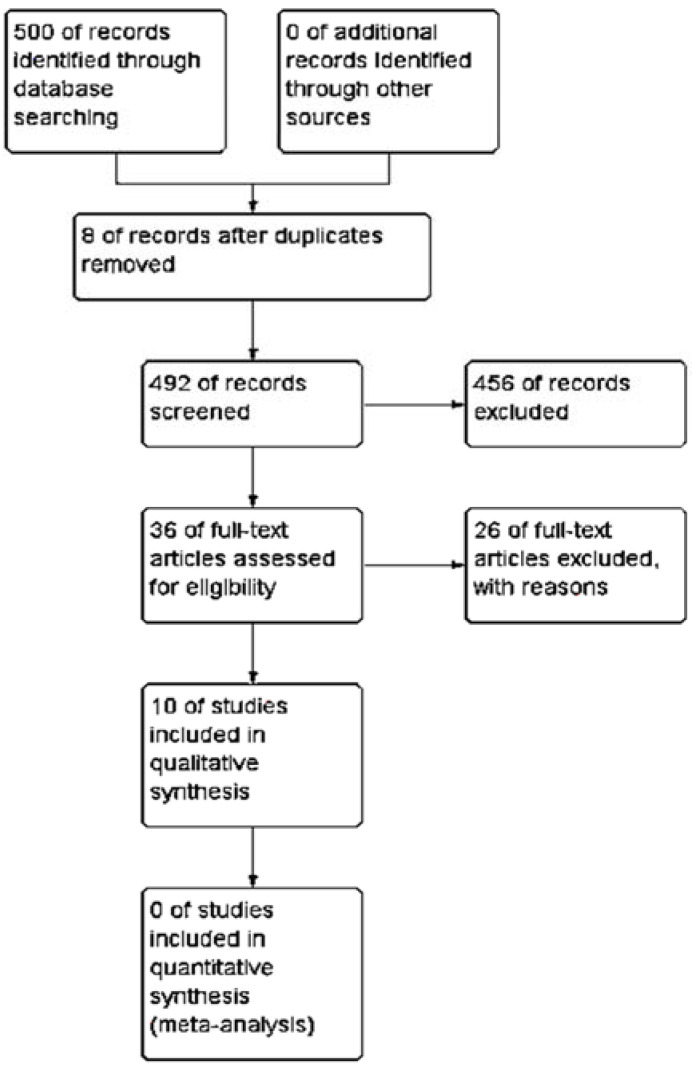
Flow diagram showing the preferred reporting items for systematic reviews and meta-analyses (PRISMA). Review Manager 5.3.

The search strategy resulted in the recovery of 500 studies (Figure 1). After the first screening, 44 studies remained, of which eight were duplicated, and 26 did not meet the eligibility criteria. Through full reading, only ten clinical trials comprised the final sample of this systematic review. The Kappa agreement index was 0.729 (*p* ≤ 0.001).

We reinforce that some of the reasons for the exclusion of some studies were wounds of periodontal and maxillary etiology, biopolymers other than platelet-rich fibrin, as well as the methodologic design, which were different from the inclusion criteria.

Therefore, Table 2 presents the selected studies with their respective references, year of publication, title, journal, and the database. Table 3 shows a summary of the clinical findings in these ten studies.

About 130 patients presented favorable events using the PRF, according to the clinical studies analyzed herein, involving 172 participants with various etiologies of wounds. Among the ten studies included, only in two clinical trials, the PRF was not superior to the outcome evaluated.

**Figure 2:**
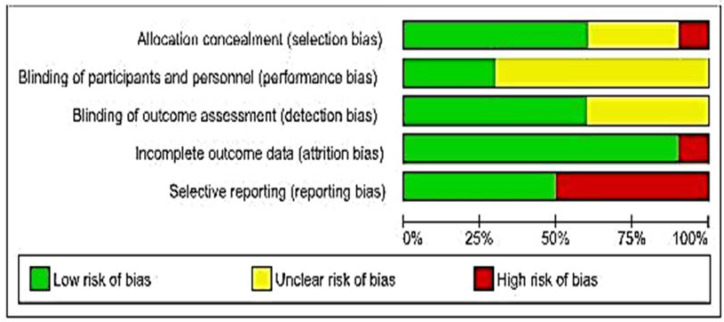
Bias Risk Summary: reviewers’ judgment for each domain and their percentages on the overall quality of the studies. (Review Manager, version 5.3).

**Figure 3:**
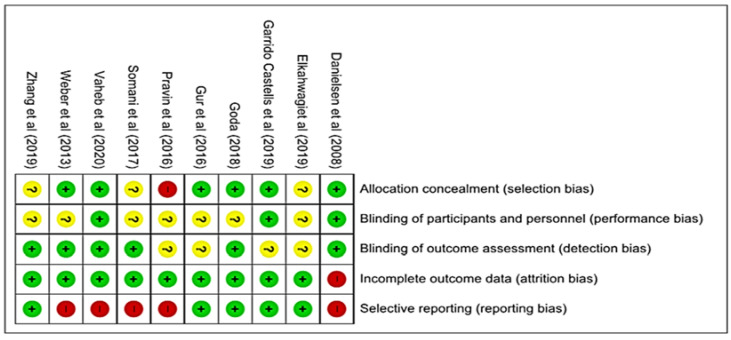
Bias Risk Summary: authors’ judgements on each item, risk bias for each included study. Review Manager, version 5.3. Legend: “+” = low risk of bias; “−“ = high risk of bias; “?” = uncertain risk of bias.

We carried out the methodological quality assessment of the ten studies included in this review using the Cochrane Collaboration tool that assesses the risk of bias in randomized clinical trials. Through the judgment of the reviewers for each domain, it was possible to infer the overall quality of the studies presented in Figure 2. The description of the results for the methodological evaluative categorization of each study is shown in Figure 3, as well as the individual judgment of the five domains. The agreement between evaluators of the risk of bias in each study was measured using the Kappa index. The value was 0.832 (*p* ≤ 0.01), which points out the credibility of the interpretation.

In the individual internal validation of the included studies, for the domain selection of bias, seven studies were classified as low risk of bias [29,30,31,34,35,38], three studies as the uncertain risk of bias [33,36,37], and one study as high risk of bias [32].

In the performance bias domain, three studies were judged as low risk of bias [26,31,35], seven studies were judged as the uncertain risk of bias [30,31,32,33,34,36,37], and no study with a high-risk rating of bias.

Regarding the detection bias domain, seven studies were classified as low risk of bias [29,30,33,34,36,38], three as the uncertain risk of bias [31,32,35,37], and no study with a high-risk rating of bias.

For the fourth evaluated domain, frictional bias, all studies were classified as low risk of bias. Finally, for the domain reporting bias, five studies were classified as low risk of bias [31,34,35,36,37], and five as high risk of bias [29,30,32,33,38].

In the critical evaluation of the studies regarding allocation secrecy, two studies were allocated to Category A [34,38], because they adequately described the allocation process; eight studies were allocated to Category B [29,30,31,33,34,35,36,37] because they did not report the allocation process of the groups effectively; however, they described the randomization process of the participants; one study allocated participants inadequately, being designated to Category C [32]. Therefore, no study was allocated to Category D. However, the majority of studies did not declare the way the randomization of individuals was carried out for the intervention and control groups in the right manner.

The performance bias of undetermined levels was observed among the analyzed studies, as they presented differences within the groups. Additionally, regardless of the intervention, there was not adequate reporting about the blinding of the participants.

The clinical trials that composed the review did not present significant losses of individuals among the groups during the proposed time (segment bias). Therefore, the analysis of the outcomes evaluated was not compromised.

The fact that most studies received funding is considered as a conflict of interest and therefore reporting bias, thus attributing to them a high risk of bias. All the studies presented significant clinical heterogeneity, making it impossible to perform a meta-analysis.

## 4. Discussion

The systematization of the treatment for wounds encompasses an approach with proper protocols for clinical evaluation and treatment management because of the barriers that interfere with healing, including the presence of necrotic tissue, senescent cells, altered extracellular matrix, hypoxia, excess of bacteria, biofilm, and inflammatory enzymes [39].

Different studies have investigated the effectiveness of healing derived from platelet concentrates. One approach is related to molecular healing strategies, which include: migration of chemotactic cytokines that facilitate the process of cell infiltration via the neutrophil-activating peptide (CXCL7), platelet factor 4 (PF4), SDF-1α (factor 1—derived from stromal cells). On the other hand, related to growth factors, it includes the platelet-derived growth factor (PDGF), epidermal growth factor (EGF), transforming growth factor (TGF-b1), vascular endothelial growth factor (VEGF) and hepatocyte growth factor (HGF). Therefore, both approaches are responsible for inducing cell proliferation and angiogenesis within chronic and acute wounds. These molecules can provide activation in different cell phenotypes. After activating the platelet concentrates, in the molecular scenario, it triggers the healing activities of different cell phenotypes. Additionally, it is also antibacterial, mainly for containing thrombocidines [40].

Autologous platelet concentrates have been an innovative approach for treating wounds of various etiologies. The PRF is widely used because it is easy to prepare and is devoid of any synthetic additives in its structure [41].

The fibrin is a protein resulting from the clotting cascade, which forms a tridimensional network, where the platelets and the immunological cells stay attached, forming a blood clotting. The platelets produce growth factors stimulating the migration of fibroblasts and the proliferation of important elements, such as collagen type I and fibronectin. Recently, the products based on fibrin have become popular for wound treatments in both hard and soft tissues, being applied in different forms, such as glue, gel, membrane, or dressings containing fibroblasts [42,43].

Although the fibrin membrane acts as a favorable scaffold to the cells, it has low mechanical resistance, and it is easily degradable. Therefore, in order to enable to support the cells for a longer period, the fibrin membrane has to be reinforced with other natural or synthetic polymers [44].

From this perspective, ten clinical trials were evaluated, including 172 acute wounds of various etiologies and segment time, which were treated with PRF from five to ten days, and chronic wounds from four to eight weeks, resulting in healing or area reduction.

Some of the studies investigated the biological effects of the PRF in acute or chronic wounds with impaired healing. Only one clinical study pointed out the benefits of using the autologous fibrin concentrate in the wound in hands and postoperative of McCash technique, presenting an acceleration of healing after a single application of PRF [42].

One of the clinical studies demonstrated no significant statistical differences in the epithelization of acute surgical wounds between the group that was conventionally treated (control group) and the group treated with PRF. However, there was less pronounced pain related by the patients from the PRF groups [29].

In addition to the acute wounds, the chronic ones provoke intense concerns, since they represent a significant and often underestimated global socioeconomic burden [44]. In the USA, about 6.5 million people are affected by chronic wounds, encouraging the development of autologous biomaterials capable of accelerating wound healing. For instance, a pilot study to evaluate the action and safety of the PRF in chronic wounds of lower limbs caused by diabetes or amputation is reported by Londahl et al. [45] as well. The results showed that the PRF accelerates the angiogenesis, creating a great area of granulation tissue in the wound bed with a reduction of the wound area in a period of treatment from eight to twenty-five days [46].

Besides the acute wounds, the chronic ones cause intense concern, since they represent a significant and sometimes underestimated global socioeconomic burden. In the USA alone, about 6.5 million people suffer from chronic wounds [45].

In this context, the PRF can be considered one of the most versatile delivery systems to the wound bed because it is an excellent carrier of growth factors and leukocytes [47].

Clinical studies reinforce the effectiveness of the PRF in the treatment of chronic wounds, especially those of venous impairment origin. In 2016, 2017 and 2018, accelerated scar processes and cures were observed in public, with the use of PRF. It is reported that the healing process is seven days faster than conventional therapy. Restoration of the tissue is described in 73.3% of the PRF group (against 53.3% of conventional treatment) [32], in 85.51% of wounds after four weeks of PRF application (against 42.74% of conventional treatment) [33], and in 100% of wounds after seven weeks of PRF application (against 42.6% of conventional treatment) [34].

Data from the clinical studies emphasize the use of PRF containing leukocytes as a simple, low-cost, fast, and easy-to-handle alternative that does not require the hospitalization of the patient. It stands out for its potential for healing and protection of soft tissues, tendons, ligaments, and bones, as well as the healing of complex chronic wounds in lower limbs [48].

The PRF can modulate the healing of soft and hard tissues through gradual and prolonged release of growth factors, guaranteeing the homeostasis and stimulating the angiogenesis and cellular proliferation [49,50]. The acceleration of the wound healing based on PRF is promoted by increasing the wound site of the growth factors, then transforming β (TGF-β), insulin-like (IGF), platelet-derived (PDGF), vascular endothelial (VEGF), fibroblastic (FGF), and epithelial (EGF) [51].

Since the autologous PRF is produced from blood, and occasionally the presence of the wound provokes blood loss, laboratory analysis was conducted by [34] to evaluate disorders in the physiology of the blood compounds compared with the blood samples from the PRF receptors. The laboratory standard exams of the hemoglobin, platelets, and albumin in the PRF group, as well as in the control group, were analyzed and compared between the groups. No statistical differences were found, reinforcing the safety of PRF production.

The use of PRF is adequate in different healing mechanisms, such as in the tympanic membrane, which presents a reversal healing cascade in the last two stages, i.e., migration and proliferation [52]. A retrospective and randomized analysis comparing the use of PRF and the conventional treatment (paper patch moistened with polyvinylpyrrolidone) of traumatic perforation of tympanic membrane demonstrated that the PRF accelerated the healing process, promoting better audiological results and removing the need for a second surgical procedure [31].

The tympanic membrane, as well as the injuries in the palatopharyngeal muscle due to the pharyngoplasty for correction of sleep apnea, do not follow the healing cascade of a sharp wound, since they are healed by the second trial, with great deposition of collagen, contraction, and granulation, followed by greater epithelization time compared with the normal healing. This kind of healing is most likely to be opened again, as there are alternative therapies to accelerate healing in this region [53]. A study contemplating the evaluation of the efficacy of the PRF to decrease the incidence of rupture of the wound in pharyngoplasty showed that the autologous PRF reduced the possibility of the wound to be opened again, and it reduced the post-surgery pain. Additionally, the patients returned to their normal diet faster than those who were conventionally treated [37].

Updated psychopathological concepts support the PRF with a dynamic multifunctional hydrogel, working as an active dressing in wound healing, responsible for releasing a large set of healing molecules, in order to be layered on the wound-bed. The degradation of PRF is highly regulated by the plasma serine protease system plasminogen activator inhibitor type 1 and 2 (PAI-1, PAI-2), TATA box-binding protein associated factor (TAF1), and plasmin, and it can be synchronized with the healing process. In contact with the wound-bed, this natural biopolymer changes its configuration and mechanical properties in conjunction with specific secondary dressings (such as hydrogels, polyurethane foams, and hydrocolloids). Furthermore, it has a well-controlled microenvironment mechanism, which is responsible for optimizing its healing activities [54,55,56].

Numerous therapies have been used to treat wounds. However, the chronicity of the injuries is a challenge for the health and biotechnology professionals because it requires the recovery of homeostasis and the recruitment of molecules to achieve healing. Although the natural hydrogels have shown favorable results in chronic wound treatments, the intrinsic properties of the fibrin matrix are superior in the acceleration of healing because of the angiogenesis stimulation [51].

The search for strategies to control the delivery of therapeutic molecules, such as growth factors, led to the emergence of sophisticated fibrin-based therapeutic delivery systems [16]. Therefore, it is necessary to develop new clinical studies and systematic reviews that may favor precise indexing protocols for targeting the peculiarities of acute and chronic wounds. 

## 5. Conclusions

The outcomes provided some evidence to support the routine using platelet-rich fibrin membrane dressings as the first line of treatment to induce the acceleration of wound healing. However, it seems that the PRF is not so efficient in the treatment of acute post-surgery wounds as in chronic wounds.

The findings do not provide sufficient evidence to support the routine use of the PRF as the first-line treatment for healing acute or chronic wounds of different etiologies. The methodological design of the majority of the clinical trials evaluated herein presented report failures, consequently affecting their results and discussion.

Based on the studies evaluated in this work, we strongly recommend the adoption of the template for intervention description and replication (TIDieR); in order to properly describe the intervention protocol, minimizing the risk of bias. We also emphasize the importance of following the directions proposed by the Consolidated Standards of Reporting Trials (CONSORT) to guarantee the reproducibility of the methodology and the adequate report. These procedures are important to accurately evaluate the benefits of the PRF applied in chronic wounds.

In this review, we found great variability in the application protocols of PRF in wounds, as well as different ways to prepare it, resulting in clinical heterogeneity. As a result, it is impossible to summarize and to classify the evidence of different types of studies to find strong conclusions.

We suggest the Intervention Description and Replication Model (TIDieR) to describe the treatment protocol, allowing for better reproducibility of the clinical trial methods, which uses PRF as an intervention for wound healing.

However, the use of PRF for the treatment of wounds of different etiologies is promising, since there is evidence in the acceleration of healing, reduction of the allergic episode, and spending on ineffective dressings. Therefore, prospective, multicenter, and large-scale clinical studies focused on short and long term therapeutic and economic impacts are necessary for the detailed implementation of this integrative practice as an alternative or adjuvant therapy for acute or chronic wounds.

## Figures and Tables

**Table 1:** Literature searching strategies.

Database	Keywords and Searching Strategies
Pubmed/MEDLINE	((“platelet-rich fibrin” (MeSH Terms) OR (“platelet-rich” (All Fields) AND “wounds” (All Fields) OR “wounds” (MeSH Terms) OR (“wound healing” (MeSH Terms) OR “wound healing” (All Fields)) AND Clinical Trial (ptyp)
EMBASE	(‘platelet-rich fibrin’/exp OR ‘platelet-rich fibrin’) AND ‘wound healing’/exp
Web of Science	TS = (Platelet-rich Fibrin * AND Wounds * OR Wound Healing *)
CENTRAL	Platelet-rich Fibrin * AND wounds * OR Wound Healing

**Table 2:** Distribution of the studies according to the authors, year of publication, title, journal, and database.

Authors/Year	Title	Journal/Database
Danielsen et al., (2008) [29]	Effect of Topical Autologous Platelet-Rich Fibrin versus No Intervention on Epithelialization of Donor Sites and Meshed Split-Thickness Skin Autografts: A Randomized Clinical Trial	Plast. Reconstr. Surg./Pubmed
Weber et al., (2012) [30]	Platelet-Rich Fibrin Matrix in the Management of Arthroscopic Repair of the Rotator Cuff A Prospective, Randomized, Double-Blinded Study	Am. J. Sports. Med./EMBASE
Gür et al., (2016) [31]	Use of a platelet-rich fibrin membrane to repair traumatic tympanic membrane perforations: a comparative study	Acta Otolaryngol./EMBASE
Pravin et al., (2016) [32]	Autologous platelet-rich plasma (PRP) versus leucocyte-platelet rich fibrin (l-PRF) in chronic non-healing leg ulcers—a randomized, open-labeled, comparative study	J. Evol. Med. Dent. Sci./EMBASE
Somani et al., (2017) [33]	Comparison of Efficacy of Autologous Platelet-rich Fibrin versus Saline Dressing in Chronic Venous Leg Ulcers: A Randomised Controlled Trial	J. Cutan. Aesthet. Surg./Web of Science
Goda (2018) [34]	Autogenous leucocyte-rich and platelet-rich fibrin for the treatment of venous leg ulcer: a randomized control study	Egypt J. Surg./Web of Science
Garrido-Castells et al., (2019) [35]	Effectiveness of Leukocyte and Platelet-Rich Fibrin versus Nitrofurazone on Nail Post-Surgery Bleeding and Wound Cicatrization Period Reductions: Randomized Single Blinded Clinical Trial	J. Clin. Med./Web of Science
Zhang et al., (2019) [36]	Platelet-rich fibrin as an alternative adjunct to tendon-exposed wound healing: A randomized controlled clinical trial	Burns/CENTRAL
Elkahwagi et al., (2019) [37]	Role of autologous platelet-rich fibrin in relocation pharyngoplasty for obstructive sleep apnoea	Int. J. Oral Maxillofac. Surg./CENTRAL
Vaheb et al., (2020) [38]	Evaluation of the Effect of Platelet-Rich Fibrin on Wound Healing at Split-Thickness Skin Graft Donor Sites: A Randomized, Placebo-Controlled, Triple-Blind Study	Int. J. Low Extrem. Wounds/CENTRAL

**Table 3:** Distribution of studies according to the number of participants, intervention group, control group, and main outcomes.

Study	Groups	Main Outcomes
[29]	Intervention group (n = 51): PRF in the incisional acute wound of laparoscopic cholecystectomy Control group (n = 51): human albumin and subcutaneous collagen deposition	The PRF in acute surgical wounds did not promote significant repairs but suppressed the synthesis and subcutaneous deposition of collagen. The study does not support the use of PRF to accelerate wound healing after surgery. However, it suggests that the PRF should be explored in the treatment of chronic wounds.
[30]	Intervention group (n = 30): PRF in acute wounds from the rotator cuff surgery Control group (n = 30): without PRF	There were no significant differences in perioperative pain, functional recovery, or structural outcomes with the use of PRF in arthroscopic repairing surgeries of the rotator cuff.
[31]	Intervention group (n = 30): PRF on the repair of the tympanic membrane perforations Control group (n = 30): paper patch, moist with polyvinylpyrrolidone 10%	The total closure of the perforations was observed in 24 (80%) patients from the PRF group and 16 (53%) from the control group (*p* < 0.05). The average improvement was 14.1 dB in the PRF group and 12.4 dB in the control group 45 days after the medical procedure (*p* < 0.05). The PRF provided faster healing than the polyvinylpyrrolidone.
[32]	Intervention group (n = 15): Platelet-rich fibrin and leukocytes (L-PRF) in chronic unhealed leg ulcers Control group (n = 15): Platelet-rich (PRP)	L-PRF had a better effect on the cure outcome of the lesion when compared to PRP. L-PRF has great anti-inflammatory effects and protects the wound against infections. At the end of the sixth application, 100% of healing was seen in 11 ulcers treated with L-PRF and eight ulcers treated with PRP (73.3% vs. 53.3%, respectively). More than 90% of improvement in the area and volume of the wounds was observed in 13 PRF cases and 10 PRP cases (86.6% vs. 66.6%).
[33]	Intervention group (n = 9): PRF in the treatment of chronic venous ulcers in legs Control group (n = 6): saline dressing	The mean reduction in the ulcer area in the PRF group was 85.51%, while in the saline group was 42.74% (*p* < 0.001). The PRF is effective, inexpensive, safe, and an outpatient procedure.
[34]	Intervention group (n = 18): PRF in the treatment of chronic venous ulcers in legs Control group (n = 18): conventional dressing	The closing rate of the wounds with initial area > 10 cm² was 50% in the sixth week and 100% in the seventh week of treatment with PRF, while in the control group was only 14.3% in the sixth week and 42.6% in the seventh one.
[35]	Intervention group (n = 20): L-PRF in post-surgical bleeding and acute wound healing in patients with bilateral onychocryptosis Control group (n = 20): use of Nitrofurazone	Statistically significant differences (*p* < 0.001) were observed between the groups showing reduction of wound healing period and post-surgical bleeding for L-PRF intervention concerning nitrofurazone treatment. L-PRF can be considered first-line supporting intervention after the surgical procedure for patients suffering from nail problems such as onychocryptosis.
[36]	Intervention group (n = 18): PRF to treat lower limb acute injury after with exposed tendons before skin grafts Control group (n = 18): treatment with dermal regeneration matrix Integra^®^	The graft acceptance rate was 92.3% in the Integra^®^ group compared to 97.83% in the PRF one (*p* < 0.001). The changes in the texture of the scar tissue were superior in the Integra^®^ group at all times in the three-months postoperative period.
[37]	Intervention group (n = 15): PRF in the postoperative acute wound, before suture, in Pharyngoplasty for treatment of obstructive sleep apnea Control group (n = 15): conventional suture	There was lower dehiscence of the wounds in the PRF group (*p* = 0.013) than in the control group. The patients from the PRF group related less pain in days 3, 5, and 10 after the surgery than those from the control group (*p* < 0.001). Additionally, the time taken to return to a normal diet was shorter in the PRF group (*p* = 0.001).
[38]	Intervention group (n = 17): PRF in burns that require a divided thickness skin graft Control group (n = 17): treatment with vaseline petrolatum gauze	The wound healing time in the PRF and control group was 11.80 ± 3.51 and 16.30 ± 4.32 days, respectively (*p* < 0.001). The PRF group presented higher rates of wound healing in days 8 and 15 compared with the control group (*p* < 0.001). There was a significant difference in average pain levels between the two groups (lower in the PRF group) (*p* < 0.001).
